# Therapeutic Efficacy of Mesenchymal Stem Cells in Modulating Oxidative Stress in Puromycin-Induced Nephropathy

**DOI:** 10.3390/pathophysiology32020019

**Published:** 2025-05-01

**Authors:** Yusuke Iizuka, Masanori Sasaki, Kojiro Terada, Takuro Sakai, Yoshinobu Nagaoka, Shinobu Fukumura, Jeffery D. Kocsis, Takeshi Tsugawa, Osamu Honmou

**Affiliations:** 1 Department of Pediatrics, Sapporo Medical University School of Medicine, Sapporo 060-8556, Hokkaido, Japan; i.yusuke@sapmed.ac.jp (Y.I.); terada.kojiro@sapmed.ac.jp (K.T.); yonagao@sapmed.ac.jp (Y.N.); tsugawat@sapmed.ac.jp (T.T.); 2 Department of Neural Regenerative Medicine, Institute of Regenerative Medicine, Sapporo Medical University School of Medicine, Sapporo 060-8556, Hokkaido, Japan; 3 Department of Neurology, Yale University School of Medicine, New Haven, CT 06510, USA; 4 Department of Perinatal Medicine, Sapporo Medical University School of Medicine, Sapporo 060-8556, Hokkaido, Japan; 5 Department of Neuroscience, Yale University School of Medicine, New Haven, CT 06510, USA; 6 Center for Neuroscience and Regeneration Research, VA Connecticut Healthcare System, West Haven, CT 06510, USA

**Keywords:** intravenous, mesenchymal stem cell, minimal change disease, puromycin aminonucleoside

## Abstract

***Background:*** Podocytes are essential for kidney function, and their dysfunction can result in nephrotic syndrome, such as minimal change disease (MCD). Oxidative stress contributes to podocyte damage. We investigated the therapeutic potential of intravenously infused mesenchymal stem cells (MSCs) in a puromycin aminonucleoside (PAN)-induced rodent MCD model, focusing on oxidative stress modulation. ***Methods:*** Sprague-Dawley rats were divided into three groups: intact, PAN-Vehicle, and PAN-MSC. MCD was induced through subcutaneous PAN injection. MSCs were infused intravenously in the PAN-MSC group on day 7. Urinary albumin, serum albumin, and creatinine levels were assessed. Histological analysis of the renal cortex was performed. Podocyte protein (NPHS1, NPHS2, and PODXL) and antioxidant enzyme (SOD1, SOD2, and GPX1) levels were measured using quantitative real-time reverse-transcription PCR (qRT-PCR). ***Results:*** MSC infusion significantly reduced proteinuria and restored podocyte structure in the PAN-MSC group. Electron microscopy revealed that infused MSCs could inhibit the fusion of the foot process induced by PAN injection. qRT-PCR showed that intravenous infusion of MSCs rescued the inhibition of GPX1 expression. GFP-labeled MSCs accumulated at the podocyte injury sites. ***Conclusion:*** Systemic MSC infusion mitigates PAN-induced MCD by reducing proteinuria, preserving podocyte structure, and modulating oxidative stress via the GPX1 pathway, offering a potential therapeutic approach for nephrotic syndrome.

## 1. Introduction

Podocytes are highly specialized, terminally differentiated epithelial cells with a critical role in renal function, and are characterized by a complex cellular architecture [1,2]. Podocyte dysfunction leads to nephrotic syndrome, including minimal change disease (MCD) [3,4]. The loss of podocyte foot processes correlates closely with the development of protein leakage across the glomerular filtration barrier, resulting in proteinuria [5,6]. While the molecular mechanisms underlying alterations in podocyte structure and function in nephrotic syndrome remain poorly understood, oxidative injury has been proposed as a possible indirect contributor to those changes [7,8]. Oxidative stress is a potential cause of podocyte damage and subsequently affects the integrity of the glomerular barrier [9,10].

Oxidative stress and reduced activity of antioxidant enzymes contribute to the development of renal diseases [11,12]. Podocyte injury induced by puromycin aminonucleoside (PAN) results in the induction of podocyte antioxidant enzyme activities [7,13]. Therefore, PAN injection in rodents is a well-described nephrosis model system for the induction of experimental nephrotic syndrome, similar to human MCD [14,15,16]. 

Cellular therapy with mesenchymal stem cells (MSCs) for MCD using the PAN injury model has been reported to have therapeutic efficacy via anti-inflammatory/immunomodulatory effects following direct injection of MSCs [14]. However, the potential role of intravenously delivered MSCs in alleviating oxidative stress in this model has not been explored.

In this study, we investigated whether intravenously infused MSCs exert a therapeutic effect on a rat MCD model caused by PAN-induced podocyte injury by an antioxidant mechanism. We performed urinalysis, blood tests, histological analysis (including light and electron microscopy), and quantitative real-time reverse-transcription PCR (qRT-PCR) on the renal cortex.

## 2. Materials and Methods

### 2.1. Preparation of MSCs from the Rat Bone Marrow

The MSC culture preparation was based on our previous studies [17]. Briefly, bone marrow obtained from femoral bones of five wild-type Sprague-Dawley (Slc: SD, Japan SLC, Shizuoka, Japan) or two green fluorescent protein (GFP)-expressing (W-Tg [CAG-GFP]184Ys, # NBRP Rat No: 0273, National BioResource Project—Rat, Kyoto University, Kyoto, Japan) rats (6–8 weeks old) was diluted with Dulbecco’s modified Eagle’s medium (DMEM; Millipore Sigma, St. Louis, MO, USA) to a volume of 15 mL and supplemented with 10% heat-inactivated fetal bovine serum (Thermo Fisher Scientific Inc., Waltham, MA, USA), 2 mM L-glutamine (Millipore Sigma), 100 U/mL penicillin, and 0.1 mg/mL streptomycin (Thermo Fisher Scientific Inc.), and was incubated for 7 days at 37 °C in a humidified atmosphere containing 5% CO_2_. When the cultures almost reached confluence, the adherent cells were detached with a trypsin-ethylenediaminetetraacetic acid solution (Millipore Sigma) and subcultured at 1 × 10^4^ cells/mL of medium. After three passages, the MSCs were used. A previous phenotypic analysis of surface antigens revealed clusters of differentiation (CD) 45^−^, CD73^+^, CD90^+^, and CD106^-^ on MSCs [18], and MSCs differentiate into mesenchymal derivatives, including osteocytes, adipocytes, and chondrocytes [19].

### 2.2. Animal Model

Male SD rats weighing 180–210 g (6-week-old) were used. The MCD was induced by PAN (#15509, Cayman Chemical, Ann Arbor, MI, USA) modified from Frenk et al. [20] and Hosoyamada et al. [21]. PAN was diluted to 20 mg/mL with saline and injected subcutaneously at 200 mg/kg body weight. All rats were maintained in a temperature-controlled environment with a 12 h light/dark cycle and were given a standard diet and water ad libitum during the study period.

### 2.3. Experimental Protocol

**Figure 1:**
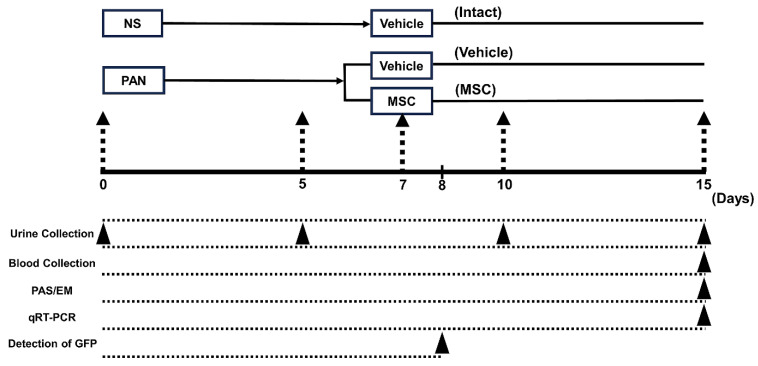
**Experimental protocol.** PAN or NS was injected subcutaneously on day 0. On day 5, PAN-injected rats received an intravenous infusion of vehicle or MSCs on day 7. Urine was collected before PAN or NS injection, and on days 5, 10, and 15 after PAN or NS injection. Blood and tissue were collected for histological analyses, and RT-PCR analyses were conducted on day 15. Tissue for detection of GFP was collected on day 8. NS, normal saline; MSC, mesenchymal stem cell; PAN, puromycin aminonucleoside; qRT-PCR, quantitative reverse-transcription PCR.

The experimental protocol is illustrated in Figure 1. The SD rats were divided into intact (n = 6), PAN-Vehicle (n = 6), and PAN-MSC (n = 6) groups. As described above, PAN was injected to induce the MCD model on day 0. For the intact group, rats were administered normal saline (NS) subcutaneously (2.0 mL/100 g body weight) on day 0. Urine samples were collected before PAN or NS injection, and at 5, 10, and 15 days after PAN or NS injection using a metabolic cage (3700M071, Tecniplast Japan Co., Ltd., Tokyo, Japan). All PAN-treated rats were confirmed to have proteinuria on day 5. On day 7, they were randomized and anesthetized with an intraperitoneal (IP) injection of ketamine and xylazine (90/4 mg/kg) and received a single intravenous infusion of MSCs at 1.0 × 10^6^ in 1.0 mL of total volume (fresh DMEM) or vehicle (1.0 mL of fresh DMEM alone) via the left femoral vein. For the intact group, rats received vehicle (1.0 mL of fresh DMEM alone) via the left femoral vein on day 7. All rats were injected subcutaneously daily with cyclosporine A (5 mg/kg), starting one day before MSC, or vehicle infusion until 15 days after injection of PAN or NS.

### 2.4. Urine Protein

To analyze 24-h urinary albumin excretion, rats were maintained in metabolic cages for 24 h every 5 days until day 15 (3700M071, Tecniplast Japan Co., Ltd.). Albuminuria was measured using enzyme-linked immunosorbent assay (n = 6/group) (LBIS™ Rat Albumin ELISA Kit, FUJIFILM Wako Pure Chemical Corporation, Osaka, Japan) [22].

### 2.5. Blood and Tissue Sample Collection

On day 15, rats (n = 6/group) were deeply anesthetized with an IP injection of ketamine (180 mg/kg) and xylazine (20 mg/kg) for euthanasia. Blood samples (2 mL) were collected from the heart. After being perfused with phosphate-buffered saline (PBS), the fresh right renal cortex was dissected for electron microscopy and RT-PCR. Then, the rats were perfused with 4% paraformaldehyde in 0.1 M phosphate buffer. The left renal cortex was dissected and post-fixed in 4% paraformaldehyde overnight. The fixed left renal cortex was embedded in paraffin for periodic acid-Schiff (PAS) staining.

### 2.6. Blood Analysis

Blood samples were centrifuged at 3000 rpm for 10 min to extract the serum and then stored at −80 °C until use. The collected serum was analyzed for serum albumin (sAlb) and serum creatinine (sCr) using DRI-CHEM NX600 (Fujifilm, Tokyo, Japan) [23].

### 2.7. PAS Staining

The 4-μm-thick sections of the paraffin-embedded left kidney were cut (RM2235, Leica, Heidelberg, Germany) and stained with PAS reagent (#40922, Muto Pure Chemicals Co., Ltd., Tokyo, Japan). PAS-stained sections were examined and photographed under a light microscope (BZ-X700; Keyence Corporation, Osaka, Japan).

### 2.8. Electron Microscopic Analysis

Dissected fresh kidneys were subjected to primary fixation with 2.5% glutaraldehyde in sodium phosphate buffer. The sections were post-fixed with 1% osmium tetroxide for 4 h, dehydrated in graded ethanol, and embedded in Epox-812 (TAAB Laboratories Equipment Ltd., Berks, UK). Ultrathin sections (70 nm) were cut using an ultramicrotome (MT-X; RMT Boeckeler Instruments, Inc., Tucson, AZ, USA) and stained with uranyl acetate and lead citrate. Ultrathin sections were examined using an electron microscope (15,000× magnification, JEM-1400, JEOL Ltd., Tokyo, Japan) operating at 80 kV. Fifty fields per animal were examined to determine the average foot process width. In each image, the curved total length of the glomerular basement membrane (BML) was measured using ImageJ (J.1.53K; Java 1.8.0_172 (64-bit), National Institutes of Health, Bethesda, MD, USA) by counting the number of slit diaphragms (Slits). The average foot process width was calculated using the formula Wp = π/4 × ΣBML/Slits [14,24].

### 2.9. RT-PCR

Quantitative RT-PCR was performed as previously described [25]. Renal cortices from each group were collected using a dissection microscope. Total RNA was extracted using an RNeasy Plus Mini Kit (#74134, Qiagen, Valencia, CA, USA) according to the manufacturer’s instructions. RNA (5 μg) was reverse-transcribed into cDNA using Superscript IV VILO Master Mix (Qiagen). Real-time PCR for each sample was performed in triplicate with TaqMan Universal Master Mix II with UNG (Thermo Fisher Scientific Inc.). The following sets of specific primers and TaqMan probes were purchased from Thermo Fisher Scientific Inc.: glyceraldehyde-3-phosphate dehydrogenase (*GAPDH*) (TaqMan rodent GAPDH control reagents, Rn01775763_g1) as endogenous control, and nephrin (*NPHS1*; Rn00575235_m1) [26], podocin (*NPHS2*, Rn00709834_m1) [26], podocalyxin (*PODXL*; Rn00593804_m1) [26], superoxide dismutase type 1 (*SOD1*; Rn01477288_m1), superoxide dismutase type 2 (*SOD2*; Rn00566942_g1), and glutathione peroxidase 1 (*GPX1*; Rn00577994_g1) [27] as target genes. The qRT-PCR analysis was performed in triplicate using PRISM7500 with 7500 software v2.3 (Thermo Fisher Scientific Inc.). Thermal cycling was conducted at 50 °C for 2 min and 95 °C for 10 min, followed by 40 cycles of 95 °C for 15 s and 60 °C for 1 min. The delta cycle threshold (Ct) (ΔCT) was calculated against the endogenous control (*GAPDH*), and the delta-delta Ct (ΔΔCT) was calculated against the ΔCT of the control. Fold change was also calculated using the comparative Ct method [28].

### 2.10. Detection of GFP-Expressing MSCs In Vivo

Six additional MCD rats were induced by the injection of PAN. The MCD rats were used to detect the infused MSCs in vivo. The MCD rats were infused with GFP-MSCs (n = 3) and non-GFP-MSCs (n = 3). GFP-MSCs were derived from GFP-expressing rats, and non-GFP-MSCs were derived from wild-type SD rats. One day after the injection of GFP-MSCs or non-GFP-MSCs, the MCD rats were deeply anesthetized with ketamine and xylazine (50/10 mg/kg, IP) and perfused with saline and 0.1 M phosphate buffer following 4% paraformaldehyde. The kidney was dissected, post-fixed in 4% paraformaldehyde overnight, cryoprotected in 30% sucrose/phosphate-buffered saline at 4 °C, embedded in OCT compound (Sakura Finetek USA, Inc., Torrance, CA, USA), and finally stored at −80 °C until use. Midcoronal sections (40 µm thickness) were prepared using a cryostat (Sakura Seiki Co., Ltd., Nagano, Japan) and mounted on glass slides. The sections were washed three times in PBS and 0.1% Tween 20, blocked in 10% normal goat serum and 0.3% Triton X-100 in PBS at room temperature for 60 min. The cryosections were processed for immunolabeling using a chicken anti-GFP antibody (1:500; ab13970, Abcam, Cambridge, UK). After washing four times in PBS-T, the sections were incubated with secondary antibodies using AF 488-conjugated goat anti-chicken IgY for GFP (1:1000; ab150169, Abcam). Furthermore, they were counterstained with 4,6-diamidino-2-phenylindole and covered with VECTORSHIELD (Vector Laboratories, Burlingame, CA, USA). The frozen sections were examined under a fluorescence microscope (BZ-X700; Keyence Corporation, Osaka, Japan).

### 2.11. Statistical Analysis

All statistical analyses were performed using JMP Pro 16 for Windows (SAS institute, Cary, NC, USA). Differences among groups were assessed using the Kruskal–Wallis test. A *p*-value of <0.05 was considered statistically significant. All data are presented as the mean ± standard error of the mean (SEM).

## 3. Results

### 3.1. Urine Albumin and Kidney Function

**Figure 2:**
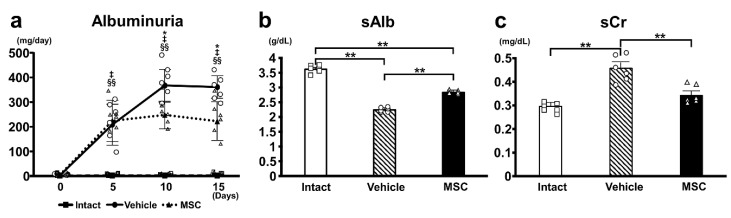
**Urine and blood analysis.** (**a**) Urine albumin was measured before PAN or NS injection and on days 5, 10, and 15 after PAN or NS injection. MSC vs. Vehicle: * *p* < 0.05, Intact vs. Vehicle ‡ *p* < 0.01, Intact vs. MSC ^§§^ *p* < 0.01. (**b**) Serum albumin (sAlb), (**c**) serum creatinine (sCr). sAlb and sCr were measured on day 15. ** *p*  <  0.01, n = 6 for each group. Error bars indicate SEM. Closed marks (■, ●, ▲) indicate average values, and open marks (□, ◯, △) indicate individual values.

Renal function was analyzed using a urinary albumin test (Figure 2a), sAlb (Figure 2b), and sCr (Figure 2c) levels. The PAN-Vehicle group induced severe albuminuria as early as day 5, which peaked on days 10–15. Intravenous infusion of MSCs (PAN-MSC group) significantly reduced albuminuria on days 10–15 compared with the PAN-Vehicle group. No urinary albumin was detected in the intact group. sAlb in the PAN-Vehicle group was significantly lower than that in the intact group; however, it was significantly greater in the PAN-MSC group than in the PAN-Vehicle group. While sCr in the PAN-Vehicle group was significantly higher than that in the intact group, it was lower in the PAN-MSC group than in the PAN-Vehicle group. These results indicated that infused MSCs reduced the deterioration of renal function.

### 3.2. Light Microscopy with PAS Staining

**Figure 3:**
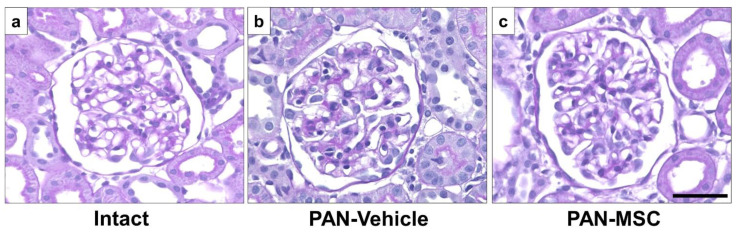
**Light microscopic analysis with PAS staining.** (**a**) Intact, (**b**) PAN-Vehicle, and (**c**) PAN-MSC groups. Scale bar = 50 μm. MSC, mesenchymal stem cell; PAN, puromycin aminonucleoside.

Histological findings of the glomeruli revealed no observable pathological features, such as focal segmental glomerulosclerosis, mesangial expansion, crescent formation, or fibrosis, in all groups using light microscopy with PAS staining. There were no differences among the intact (Figure 3a), PAN-Vehicle (Figure 3b), and PAN-MSC (Figure 3c) groups.

### 3.3. Electron Microscopy

**Figure 4:**
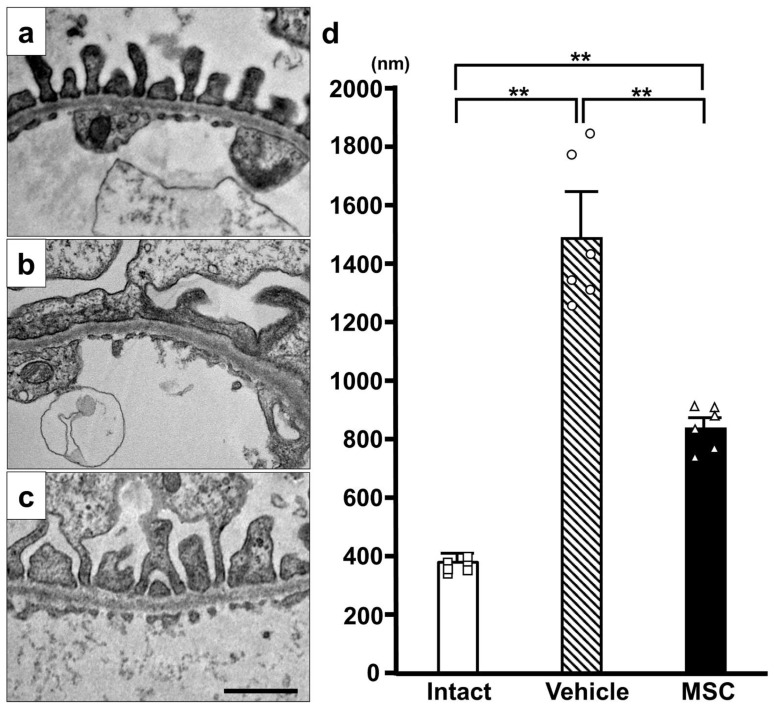
**Electron microscopic analysis.** Representative electron microscopy images of (**a**) Intact, (**b**) PAN-Vehicle, and (**c**) PAN-MSC groups. (**d**) Quantification of podocyte foot width. Scale bar = 1 μm. ** *p*  <  0.01, n = 6 for each group. Error bars indicate SEM. Open marks (☐, ◯, △) indicate individual values.

Electron microscopy showed normal podocyte morphology in the intact group (Figure 4a). However, podocytes in the PAN-Vehicle group (Figure 4b) showed extensive effacement of the foot processes without detachment, which is a characteristic of MCD. The conventional characteristic of podocyte effacement is widening of the foot processes and a decrease in the length of the slit diaphragm per glomerular basement membrane area [29]. The PAN-MSC group (Figure 4c) showed less effacement of the foot processes. The quantification of the podocyte foot width on day 15 (Figure 4d) showed that the podocyte foot width in the PAN-Vehicle group was wider than that in the intact group. The podocyte foot width in the PAN-MSC group was shorter than that in the PAN-Vehicle group. There were no differences between the intact and PAN-MSC groups. These results indicate that infused MSCs may inhibit the foot process fusion induced by PAN injection.

### 3.4. Expression of Podocyte-Associated Proteins

**Figure 5:**
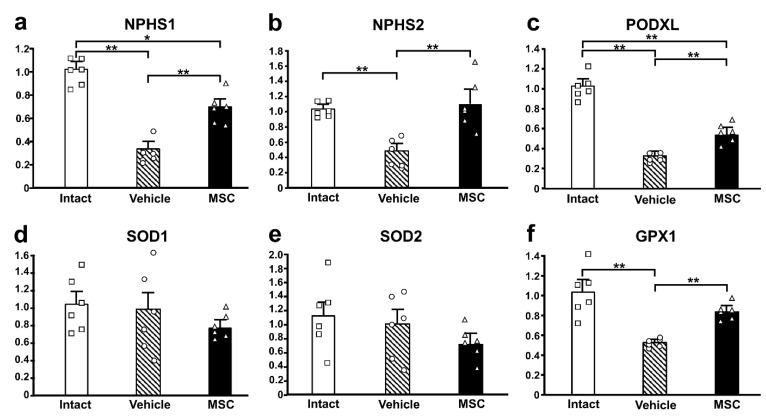
**Gene expression of podocyte-associated and oxidative stress-related genes.** Relative mRNA expression levels of (**a**) *NPHS1*, (**b**) *NPHS2*, (**c**) *PODXL*, (**d**) *SOD1*, (**e**) *SOD2*, and (**f**) *GPX1*. * *p*  <  0.05, ** *p* < 0.01, n = 6 for each group. Error bars indicate SEM. Open marks (☐, ◯, △) indicate individual values.

To investigate the podocyte-associated proteins in the current study, we performed qRT-PCR to assess the mRNA expression levels of *NPHS1* (Figure 5a), *NPHS2* (Figure 5b), and *PODXL* (Figure 5c). The mRNA expression of these three genes was downregulated in the PAN-Vehicle group compared with the intact group. Meanwhile, the mRNA expression of three genes was upregulated in the PAN-MSC group compared with the PAN-Vehicle group. The mRNA expression of *NPHS1* and *PODXL* was higher in the intact group than in the PAN-MSC group. There were no differences in *NPHS2* between the intact and PAN-MSC groups. These results suggest that infused MSCs may contribute to restoring podocyte damage induced by the PAN injection.

### 3.5. Antioxidant Enzyme Activity

To examine the activity of antioxidant enzymes in this study, the mRNA expression of *SOD1* (Figure 5d), *SOD2* (Figure 5e), and *GPX1* (Figure 5f) was quantified. There were no differences between the three groups in the expression of *SOD1* and *SOD2*. However, the mRNA expression of *GPX1* was downregulated in the PAN-Vehicle group compared with the intact group. The expression levels in the PAN-MSC group were not downregulated compared with the intact group, and the expression levels in the PAN-MSC group were higher than those in the PAN-Vehicle group.

### 3.6. Detection of GFP-MSCs

**Figure 6:**
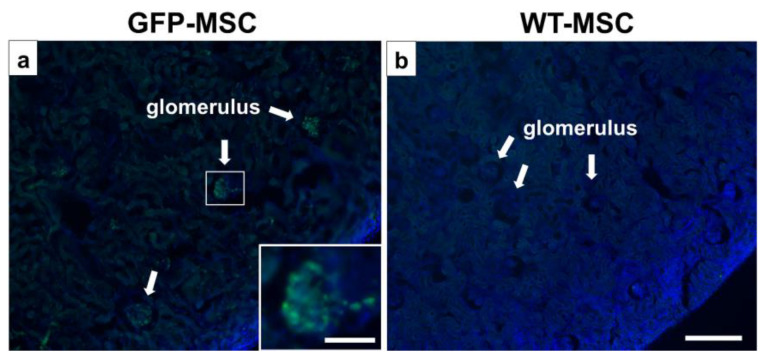
**Distribution of GFP-MSCs in vivo.** The intravenously infused GFP-MSCs (green) were observed in the DAPI (blue)-counterstained glomerulus (**a**) 1 day after GFP-MSC administration. (**b**) No GFP-positive signals in the non-GFP-MSC (WT-MSC)-infused MCD rats. Scale bar = 300 μm (**a**,**b**) and 100 μm (inset in a). DAPI, diamidino-2-phenylindole; GFP, green fluorescent protein; MCD, minimal change disease; MSC, mesenchymal stem cell; WT, wild type.

GFP signals were detected in the glomerulus by observing green fluorescence (Figure 6a), indicating that GFP-MSCs may accumulate at the podocyte injury site. To rule out the possibility of autofluorescence at the GFP detection wavelength, we examined tissue sections from PAN-treated rats infused with non-GFP-MSCs derived from wild-type SD rats. No GFP^+^ cells were observed in these control rats (Figure 6b).

## 4. Discussion

In this study, we aimed to elucidate the therapeutic mechanism of MSCs in a rodent model of MCD induced by PAN injection through subcutaneous administration of PA (200 mg/kg body weight), mimicking the clinical and pathological features of human nephrotic syndrome. PAN is a classic and well-established nephrotoxic agent [30]. The PAN-injected rats displayed deteriorated renal function and extensive effacement of the foot processes of podocytes, indicating the establishment of MCD, which was consistent with the findings of previous studies [14]. Intravenous infusion of MSCs provided therapeutic efficacy, as evidenced by improvement in renal function and podocyte structure. Gene expression analysis revealed that the podocyte-associated proteins (*NPHS1*, *NPHS2*, and *PODXL*) were downregulated following PAN injection, and their expression was maintained in the PAN-MSC group similar to the intact group, indicating a protective effect of MSCs at the molecular level.

To elucidate the role of oxidative stress in this model system, we examined the mRNA expression of key antioxidant enzymes. The mRNA expression levels of SOD1 and SOD2 did not show significant differences among the three groups. It is possible that PAN-induced nephrotoxicity in the current study was not strong enough to change the mRNA expression of SOD1 and SOD2 [31]. However, the mRNA expression level of GPX1 was downregulated in the PAN-Vehicle group compared with the intact group [32]. Taken together, these findings indicate that while the expression levels of SOD1 and SOD2 remained unchanged, only the expression of GPX1 was affected. This unique pattern of antioxidant enzyme expression observed in this study could contribute to identifying the possible action site of MSCs. The alteration in GPX1 alone suggests that infused MSCs may potentially influence GPX1 activity in podocytes. Further studies are needed to fully elucidate the therapeutic mechanisms after infusing MSCs in PAN-induced podocyte injury related to oxidative stress.

The rat model of MCD induced by PAN administration was established with stable expression of SOD and downregulation of GPX1 activity. GPX1 is an intracellular antioxidant enzyme that uses reduced glutathione to convert H_2_O_2_ to water to limit its harmful effects [33]. Downregulated expression of GPX1 induced podocyte injury due to excessive H_2_O_2_ accumulation. Accumulation of H_2_O_2_ exerts podocyte injury [33]. Infused MSCs in this study inhibited the downregulation of mRNA expression of GPX1 in the PAN-MSC group, which may reduce excessive H_2_O_2_. This might reduce the damage to podocytes in the PAN-MSC group. We observed improved renal function with restored GPX1 following intravenous infusion of MSCs in this model. Thus, the beneficial effects on kidney function through the protection of GPX1 expression by intravenous infusion of MSCs might be a novel finding in the current study.

Ornellas et al. (2019) employed a severe form of the podocyte injury model induced by two doses of PAN with unilateral nephrectomy [14]. Direct injection of MSCs under the kidney capsule in the model resulted in renal protection. Injected MSCs induced the downregulation of proinflammatory Th1 cytokines with a shift to an increase in regulatory Th2 cytokines associated with increased vascular endothelial growth factor expression in the kidney, suggesting immunomodulatory effects by injected MSCs [14]. Since oxidative stress has been identified as a key contributing factor to inducing PAN-mediated podocyte injury [34,35], we investigated whether infused MSCs are related to the modulation of oxidative stress. We found that infused MSCs play an important role in improving renal function in a PAN-induced podocyte injury via the GPX1 mechanism.

This study provides evidence that intravenously infused MSCs can mitigate the effects of PAN-induced MCD in a rodent model by modulating oxidative stress and improving renal function. Accumulated GFP^+^-MSCs were found in the glomeruli, with few detectable fluorescence signals in the other renal areas. This suggests a tropism of MSCs toward the injured area. Indeed, we previously reported the accumulation of MSCs in injury areas in various disease models [17,25]. The preservation of GPX1 expression, coupled with consistent levels of SOD1 and SOD2, suggests an enzyme-specific antioxidant response that is effectively facilitated by intravenous infusion of MSCs. These findings highlight the potential of infused MSCs as a promising therapeutic approach for nephrotic syndrome and provide a novel mechanism by which MSCs exert their protective effects via oxidative stress pathways. Future studies should elucidate the detailed molecular interactions between MSCs and podocytes, especially those involved in oxidative stress pathways, and their interplay with other relevant signaling mechanisms, including anti-inflammatory pathways. The insights gained from this study could pave the way for the development of advanced MSC-based therapies that could significantly improve outcomes for patients with nephrotic syndrome and other related kidney diseases. Finally, although prednisone was not used in the current study, it could be incorporated into future studies to explore potential additive or synergistic effects.

## 5. Conclusions

This study demonstrates that intravenously infused MSCs significantly improve renal function and podocyte structure in a rodent model of PAN-induced MCD. Infused MSCs reduced proteinuria, preserved podocyte foot processes, and maintained the expression of key podocyte-associated proteins. The therapeutic effects are likely due to the modulation of oxidative stress, as evidenced by the protection of GPX1.

## Data Availability

The datasets used and/or analyzed during the current study are available from the corresponding author upon reasonable request.
